# Land use and land cover data changes in Indian Ocean Islands: Case study of Unguja in Zanzibar Island

**DOI:** 10.1016/j.dib.2017.01.010

**Published:** 2017-01-17

**Authors:** Sizah Mwalusepo, Eliud Muli, Asha Faki, Suresh Raina

**Affiliations:** a icipe - African Insect Science for Food and Health, P.O. Box 30772-00100, Nairobi, Kenya; b Department of General Studies, Dares Salaam Institute of Technology, P.O. Box 2958, Dares Salaam, Tanzania; c South Eastern Kenya University, P.O. BOX 170-90200, Kitui, Kenya; d Agricultural Sector Development Programme, P.O. Box 159, Zanzibar

**Keywords:** Landscape ecology, Land use/cover, Geographical Information Systems tool, Unguja Island

## Abstract

Land use and land cover changes will continue to affect resilient human communities and ecosystems as a result of climate change. However, an assessment of land use and land cover changes over time in Indian Ocean Islands is less documented. The land use/cover data changes over 10 years at smaller geographical scale across Unguja Island in Zanzibar were analyzed. Downscaling of the data was obtained from SERVIR through partnership with Kenya-based Regional Centre for Mapping of Resources for Development (RCMRD) database (http://www.servirglobal.net), and clipped down in ArcMap (Version 10.1) to Unguja Island. SERVIR and RCMRD Land Cover Dataset are mainly 30 m multispectral images include Landsat TM and ETM+Multispectral Images. Landscape ecology Statistics tool (LecoS) was used to analysis the land use and land cover changes. The data provide information on the status of the land use and land cover changes along the Unguja Island in Zanzibar. The data is of great significance to the future research on global change.

**Specifications Table**

**Table:** 

Subject area	Environmental science
More specific subject area	Land use and Land cover changes analysis
Type of data	Tables and Figures
How data was acquired	Data was acquired by downscaling on 30 m Land Cover Dataset from SERVIR and RCMRD database.
Data format	Analyzed
Experimental factors	We make use of Regional Centre for Mapping of Resources for Development (RCMRD) and SERVIR database.
Experimental features	Landscape ecology Statistics tool (LecoS) was used to analysis the changes and Geographic Information System GIS software (ArcMap version 10.1) to generate maps showing the spatially continuous data over the study area.
Data source location	Landsat data, historical maps, and auxiliary data
Data accessibility	Data are available in this article

**Value of the data**

• The data provide information on the status land use and land cover changes across Unguja Island.
• The data are important in environmental assessments and against the impact of climate changes due to emissions of green house gases.
• The data can be used for modeling the effects on pollinators in Indian Ocean Islands, in particular Zanzibar Island.
• The data is valuable for improvements computational facilities, insufficient land use/land cover and reliable downscaling at smaller geographical scale.
• The data are important for agriculture, settlement, urban planning, researcher, scholar and academics.

## 1 Data

**Fig. 1:**
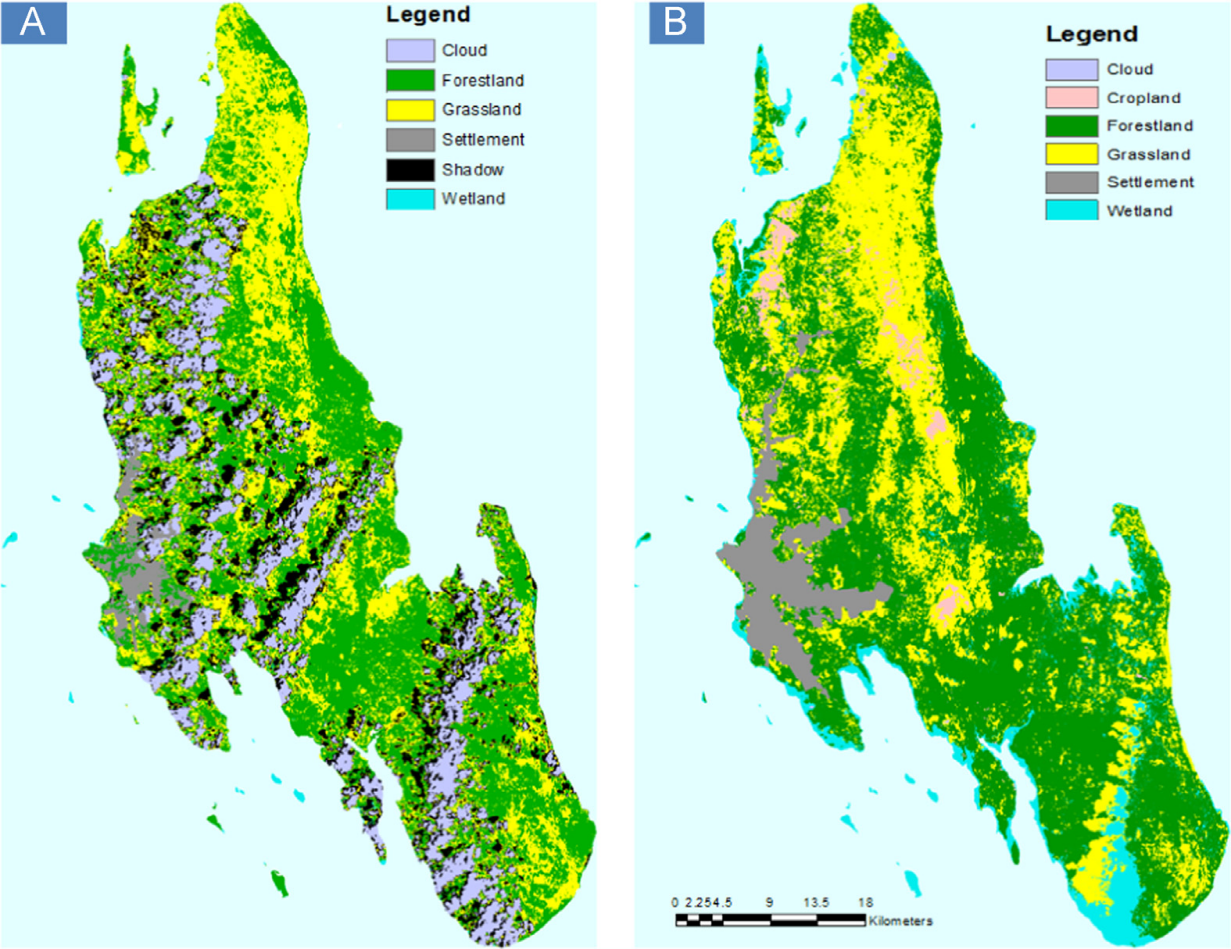
Land use/cover of Unguja Island; (A) classification scheme one for the year 2000 and (B) classification scheme one for the year 2010.

**Fig. 2:**
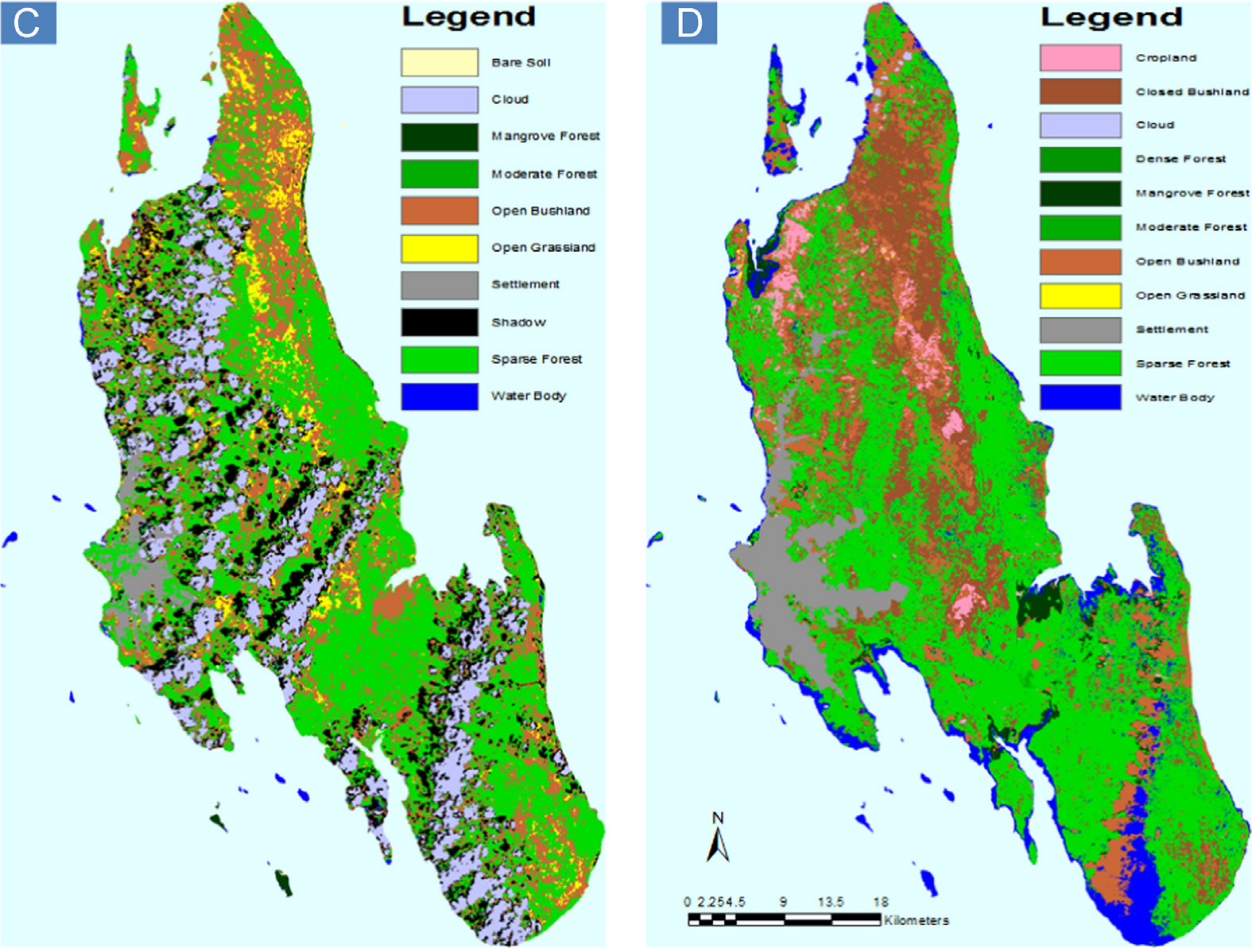
Land use/cover of Unguja Island; (A) classification scheme two for the year 2000 and (B) classification scheme two for the year 2010.

Fig. 1, Fig. 2 show the distribution of the main land use/land cover data, drawing on the databases for the period 2000 and 2010. Fig. 1 shows spatially continuous data on land use/cover classification scheme one, with six land use/cover types. Fig. 2 shows spatially continuous data on land use/cover classification scheme two, with 10 land use/cover types. These are followed by Table 1, Table 2, respectively. The tables show the percentage of land use/land cover changes in Unguja Island over categories over time. From Tables and Figures, it is apparent that there have been changes in the land use and land cover types across Unguja Island. Forestland has been reducing in area coverage over time (Table 1), and it is evident enough on the maps (Figs. 1B and 2D) that forest has been converted to settlement and agriculture, probably due to population pressure, poverty, and unemployment.

## 2 Experimental design, materials and methods

In brief, the study site is localized in Zanzibar Island in Eastern and South Eastern coast of Africa. The Island is a semi-autonomous archipelago in Tanzania, but it has an autonomous administrative government for matters that are not part of the union government. The target area was Unguja Island and is located between latitude 6°08′ and Longitude 39°20′E.

Our dataset was obtained from Regional Centre for Mapping of Resources for Development (RCMRD)-SERVIR database (http://www.rcmrd.org or https://www.servirglobal.net). RCMRD-SERVIR has verified the land use/land cover maps through ground verification campaigns, and baseline data are provided in the form of Landsat satellite imagery, auxiliary data and as well as historical maps. The classification systems includes forestland, grassland, settlement, shadow, wetland, water bodies, cloud, cropland, bare soil, mangrove forest, dense forest, sparse forest, moderate forest, open grassland, open bushland, and closed bushland were considered. Classification scheme one (includes only six land use/cover types) and classification scheme two (includes only ten land use/cover types) for the year 2000 and 2010 were used for analysis. Landscape ecology statistics tool (LecoS) [1], [2] was used to analysis the changes over time and Geographic Information System GIS software (ArcMap version 10.1) to generate maps showing the spatially continuous data across the study area.

## Figures and Tables

**Table 1:** Percentage of land use/ land cover changes in Unguja Island over period 2000–2010 for classification scheme one.

					Changes	
	2000		2010		2000–2010	
LULC Class	Area (ha)	Relative coverage (%)	Area (ha)	Relative coverage (%)	Area (ha)	Relative coverage (%)
Forestland	25364474.1	27.5	25036755.3	28.9	−327718.7	−1.3
Grassland	50188970.9	54.4	44948731.4	51.8	−5240239.6	−10.4
Wetland	15713158.4	17.0	16048430.8	18.5	335272.4	+2.1
Settlement	130037	0.1	227756.9	0.3	97719.9	+75.1
Cloud	843719.6	0.9	495397.1	0.6	−348322.5	−41.3
Total area (ha)	92240360.1		86757071.5			

Note: Positive sign means increase while negative sign means decrease in area.

**Table 2:** Percentage of land use/land cover changes in Unguja Island over period 2000–2010 for classification scheme two.

					Changes	
	2000		2010		2000–2010	
LULC Class	Area (ha)	Relative coverage (%)	Area (ha)	Relative coverage (%)	Area (ha)	Relative coverage (%)
Moderate Forest	3084209.5	4.0	3585289.7	5.0	501080.22	+16.2
Sparse Forest	23008872.42	29.9	20767214.7	29.2	−2241657.7	−9.7
Mangrove Forest	79915.8	0.1	134590.7	0.2	54675.09	+68.4
Open Grassland	9926227.9	12.9	8640365.3	12.1	−1285862.6	−13.0
Open Bushland	24745333.3	32.2	22331228.9	31.4	−2414104.4	−41.3
Water Body	15068977.6	19.6	15046558.7	21.1	−22418.8	−9.8
Settlement	134414.7	0.2	227756.9	0.3	93342.2	+69.4
Cloud	847370.3	1.1	495397.1	0.7	−351973.3	−41.5
Total area (ha)	76895321.9		71228402.2			

Note: Positive sign means increase while negative sign means decrease in area.

## References

[1] Steiniger S., Hay G.J. (2009). Free and open source geographic information tools for landscape ecology. Ecol. Inform..

[2] Wu J. (2006). Landscape ecology, cross-disciplinarity, and sustainability science. Landsc. Ecol..

