# Supplementary material for: Land use and land cover data changes in Indian Ocean Islands: Case study of Unguja in Zanzibar Island
Source: Data Brief. 2017 Jan 17;11:117–21. doi: 10.1016/j.dib.2017.01.010 (PMC5288318; doi:10.1016/j.dib.2017.01.010)
Supplement: Supplementary file 1 — Supplementary material [file mmc1.doc]

Editorial Board for Journal of Data in Brief

Manuscript Draft

Manuscript Number:

Title: **Land use and Land cover data changes in Indian Ocean Islands: Case study of Unguja in Zanzibar Island**

Article Type: Data Article

**Keywords**: Landscape ecology, Land use/cover, Geographical Information Systems tool, Unguja Island

**Corresponding Author**: Sizah Mwalusepo

***E-mail address*:** [mwalusepo@yahoo.com](mailto:mwalusepo@yahoo.com)/[smwalusepo@icipe.org](mailto:smwalusepo@icipe.org)

***Mobile phone***: +254700459981/+255754864493

**First Author**: Sizah Mwalusepo

**Order of Authors**: Sizah Mwalusepo, Eliud Muli

**Abstract**: Land use and land cover changes will continue to affect resilient human communities and ecosystems as a result of climate change. However, an assessment of land use and land cover changes over time in Indian Ocean Islands is less documented. The land use /cover data changes over 10 years at smaller geographical scale across Unguja Island in Zanzibar were analyzed. Downscaling of the data was obtained from SERVIR through partnership with Kenya-based Regional Centre for Mapping of Resources for Development (RCMRD) database (http://[www.servirglobal.net](http://www.servirglobal.net/)), and clipped down in ArcMap (Version 10.1) to Unguja Island. SERVIR and RCMRD Land Cover Dataset are mainly 30 meter maltispectral images include Landsat TM and ETM + Multispectral Images. Landscape ecology Statistics tool (LecoS) was used to analysis the land use and land cover changes. The data provide information on the status of the land use and land cover changes along the Unguja Island in Zanzibar. The data is of great significance to the future research on global change.

**Conflicts of interest**

December 14, 2016.

Editor-in-Chief

Dear Editor:

Enclosed for your consideration is an original research article, entitled “**Land use and Land cover data changes in Indian Ocean Islands: Case study of Unguja in Zanzibar Island”,** which we submit for publication in the Journal of Data in Brief. Here, I declare that:

1. All authors of this research paper have directly participated in the planning, execution, or analysis of this study.
2. The contents of this manuscript have not been copyrighted or published previously.
3. The contents of this manuscript are not now under consideration for publication elsewhere.
4. The contents of this manuscript will not be copyrighted, submitted, or published elsewhere, while acceptance by the Journal is under consideration.
5. There are no directly related manuscripts, published or unpublished, by any authors of this paper.
6. The paper contain only two figures and two tables

We think this work bring a valuable contributions to the understanding land use and land cover dataset at a smaller geographical scale in Zanzibar Island. Hence we hope that these interesting dataset make of this study candidate of interest for your journal.

Sincerely yours,

Sizah Mwalusepo (on behalf of all co-authors)
